# Determinants of neonatal near miss among newborns admitted to SOS Mother & Child Hospital, Benadir region, Somalia: a case-control study

**DOI:** 10.1186/s40748-025-00234-7

**Published:** 2025-11-05

**Authors:** Hassan Abdullahi Dahie, Falis Ibrahim Mohamud, Mohamed Abdullahi Osman, Yusuf Ali Jimale, Hamdi Ahmed Hussein, Mohamed Osman Alasow, Abukar Abdi Osman, Abdirahman Mohamed Abdullahi, Mohamed Maalin Dakane, Dek Abdi, Abdullahi Adan Isak, Lukman Sheikh Omar, Bashir Said Hassan, Sadia Hussein Mohamud, Abdihakin Mohamed Hassan

**Affiliations:** SOS College of Health Science University, SOS Children’s Villages, Mogadishu, Somalia

**Keywords:** Neonatal near miss, Somalia, Determinants, Antenatal care, Neonatal outcomes, Maternal health, Case-control study

## Abstract

**Background:**

While the birth of a newborn is often a moment of great joy, it can be overshadowed by life-threatening complications that endanger survival in the early days of life. Neonatal near-miss (NNM) cases are infants who survive severe complications, offer a valuable lens for evaluating the quality of neonatal care. Somalia continues to have one of the highest neonatal mortality rates globally, with about 37 deaths per 1,000 live births, highlighting significant gaps in maternal and child health services. This study aimed to identify the determinants of neonatal near miss among neonates admitted to SOS Mother & Child Hospital, Banadir, Somalia.

**Methods:**

An unmatched case-control study was conducted at SOS Mother and Child Hospital in Banadir region from December 2024 to April 2025. A total of 243 neonatal near miss (NNM) cases and 730 healthy neonate controls were included. Cases were identified using pragmatic and management criteria from the Centro Latinoamericano de Perinatología (CLAP) criteria. For each case, three controls were randomly selected. Data were collected using structured questionnaire and record reviews and analyzed using SPSS v25. Logistic regression was employed to identify independent predictors of neonatal near miss.

**Results:**

Significant predictors of neonatal near miss included lack of maternal (aOR: 2.61) and paternal education (AOR: 3.64), monthly household income below 100 USD (aOR: 2.82), short birth interval under 24 months (aOR: 1.97), lack of antenatal care (ANC) attendance (aOR: 6.25), history of stillbirth (aOR: 4.35), obstetric complications (aOR: 4.46), preterm or post-term birth (AOR: 1.89), prolonged labor (aOR: 3.58), home delivery (aOR: 4.76), maternal chronic illness (aOR: 3.37), male sex of the newborn (aOR: 1.86), and low birth weight (aOR: 9.34).

**Conclusion & recommendation:**

Neonatal near miss remains a pressing public health concern in Somalia, influenced by socio-demographic, obstetric, and neonatal factors. Strengthening maternal education, promoting antenatal care, ensuring skilled birth attendance, and improving facility-based delivery services are essential to reducing neonatal complications and improving outcomes. Policymakers and humanitarian partners must prioritize investments in maternal and newborn health to address these preventable risks.

## Background

The arrival of a neonate evokes profound love and anticipation, as families eagerly await their beloved new addition [1]. However, this joyous occasion may sometimes be overshadowed by concerns stemming from critical life-threatening challenges for both the mother and the neonate. During this journey, many infants tragically lose their lives, while others experience near-miss events, narrowly escaping severe health complications.

Annually, 135 million newborns enter the world, commencing their journey in an identical state of vulnerability [2]. Yet, their prospects for survival and flourishing diverge markedly depending on the global context of their birth [3]. This spectrum ranges from high-income nations equipped with universal neonatal intensive care to environments where births occur at home, devoid of midwifery support, medical provisions, or institutional healthcare assistance [4].

Globally, 2.3 million children died in the first month of life in 2022, equating to approximately 6,300 neonatal deaths every day [5]. Sub Saharan Africa accounted for 57% of these deaths, despite only having 30% of global live births. This disparity resulted in the region having the highest neonatal mortality rate in the world, with 27 deaths per 1,000 live births [6]. Somalia remains one of the countries with the highest neonatal mortality rates worldwide, with approximately 37 deaths per 1,000 live births, reflecting substantial gaps in maternal and child health services [7].

Although neonatal mortality is an indicator of neonatal health, it only reveals the tip of the iceberg, as most neonates who survive complications remain unnoticed [3]. Understanding the full extent of neonatal ill-health requires studying neonates who survived from severe complications (neonatal near miss) in addition to neonatal deaths [8]. Neonatal near miss is defined as a condition of newborn infant characterized by severe morbidity (near miss) of pragmatic and management criteria but survived these conditions within the first 28 days of life [9]. Like the concept of maternal near miss, neonatal near miss (NNM) is gaining prominence as an emerging methodology and is increasingly recognized as a critical indicator for evaluating the quality of neonatal care [10–12]. This approach plays a pivotal role in efforts to mitigate preventable neonatal morbidity and mortality.

Somalia experiences one of the highest neonatal mortality rates globally, with 37 deaths per 1,000 live births, reflecting significant public health challenges. This crisis is compounded by limited access to quality healthcare and inadequate maternal health services, as only 24% of pregnant women receive at least four antenatal care visits, and just 32% of deliveries are attended by skilled birth attendants [13, 14]. Prolonged conflict and instability have further weakened the country’s health infrastructure, leaving maternal and child healthcare services severely under-resourced and unable to meet the growing needs of the population [15]. Globally, neonatal near miss (NNM) prevalence remains a significant concern, with survival rates among newborns with severe complications ranging from 37.1 to 45.3%. In Africa, the pooled prevalence of NNM is approximately 30%, though this figure varies across regions and time periods [7, 16]. This relatively lower prevalence in Africa can be attributed to the high neonatal mortality rates, where many sick neonates do not survive due to limited healthcare access and resources. Data on NNM in Somalia remains scarce; however, studies from the Banadir region identify critical factors contributing to NNM, such as inadequate antenatal care, short birth intervals, and low maternal education [17]. Therefore, the study examined the determinants of neonatal near miss among neonates born in major public hospitals in the Banadir Region, Somalia.

## Methods

### Study design

This was a hospital-based unmatched case-control study designed to assess neonatal near-miss events by comparing affected neonates (cases) with healthy neonates (controls).

### Setting

The study was conducted at SOS Mother and Child Hospital in the Banadir region, Somalia, from December 2024 to April 2025. This facility is a major maternal and child health hospital, offering comprehensive neonatal care with approximately 200 beds and admitting an average of 65 newborns monthly.

### Participants

The study participants consisted of neonates admitted to SOS Mother and Child Hospital during the study period, classified into two groups: cases and controls. Cases included neonates who experienced at least one neonatal near-miss event within the first 27 days of life but ultimately survived. Identification of neonatal near-miss cases was based on criteria established by the Latin American Centre for Perinatology (CLAP), which include both pragmatic and management criteria. Pragmatic criteria were neonates born weighing less than 1750 g, delivered before 33 weeks of gestation, or with an Apgar score of less than 7 at five minutes. Management criteria encompassed neonates who required critical clinical interventions such as therapeutic antibiotics, nasal continuous positive airway pressure (NCPAP), neonatal intubation, phototherapy initiated within 24 h of birth, cardiopulmonary resuscitation, administration of vasoactive medications, anticonvulsants, surfactants, blood products, steroids for refractory hypoglycaemia, or surgical procedures during their neonatal period [10, 11].

Controls were healthy neonates selected from the hospital’s postnatal wards. These neonates were born without complications, did not require any specialized medical interventions, and were discharged in stable condition. To enhance comparability, for every neonate identified as a near-miss case, three control neonates were carefully selected on the same day the near-miss event occurred [18].

Neonates were excluded from the study if they had an unknown or incomplete birth history, belonged to multiple gestations, experienced maternal absence during admission, possessed incomplete medical records, or were initially categorized as controls but subsequently reclassified as cases during the study period [19].

### Sampling procedures

Cases were selected using consecutive sampling. All neonates admitted to the newborn unit who met the neonatal near-miss (NNM) criteria during the study period were included as cases at the time of discharge. This ensured that every eligible case was captured without omission until the required sample size was achieved. For each case included in the study, three controls were randomly selected from among neonates who were born healthy and discharged without complications. A list of all eligible healthy neonates admitted to the postnatal ward was compiled using their registration numbers. From this list, controls were selected using computer-generated simple random sampling, maintaining a 1:3 case-to-control ratio.

### Sample size determination

The sample size for this study was calculated using the double population proportion formula through the Epi Info 7 StatCalc program. The calculation was based on the following assumptions: a 95% confidence level, 80% power, and a case-to-control ratio of 1:3. The percentage of cases exposed to lack of ANC (12%) and the percentage of controls exposed to lack of ANC (5.7%) were taken from a study conducted in Northeast Ethiopia [20]. Based on these parameters, the required sample size was calculated to be 824 participants. After adjusting for an 18% non-response rate, the final sample size increased to 973 participants (243 cases and 730 controls).

### Data collection method

Data was collected from the mothers of the neonates using a structured and pretested questionnaire, administered by trained interviewers. The questionnaire was carefully adapted from relevant literature to ensure its validity and relevance [19, 21, 22]. The data collectors used face-to-face interviews and client record review techniques to collect data. Structured interviews with mothers of newborns were conducted in private settings, primarily at the time of patient discharge, ensuring that cases had recovered from their illnesses and controls were physiologically stable and ready for discharge. In addition to interviews, relevant clinical data were extracted from hospital records with appropriate permissions. This dual approach enhanced data richness by providing objective clinical information alongside maternal reports.

### Study variables

Neonatal near miss was defined as a neonate who experienced a severe, life-threatening condition during the neonatal period (0–28 days) but survived, as per established clinical or management criteria. This outcome variable was coded as 1 = ‘Yes’ and 0 = ‘No’.

Independent variables included socio-demographic characteristics such as maternal age, education and occupation of both parents, marital status, household size, income, and residence. Obstetric history covered gravidity, parity, previous stillbirths, abortions, neonatal deaths, and birth intervals.

Maternal health conditions during pregnancy such as anemia, hypertension, diabetes, heart disease, and infections were considered, along with health service factors like ANC attendance, number of visits, and complications during labor and delivery.

Newborn-related variables included sex, gestational age, birth weight, APGAR score, and presentation. Critical interventions such as use of antibiotics, CPAP, intubation, phototherapy, CPR, and presence of congenital anomalies were also assessed to identify near-miss cases.

### Data quality control

The data were collected by ten qualified midwives fluent in both English and Somali. Prior to data collection, they received an intensive two-day training that covered data collection procedures, the objectives of the study, questionnaire content, participant interaction, and ethical considerations such as confidentiality and privacy.

One week before data collection began, a pretest was conducted on 5% of the sample to assess the clarity, validity, and usability of the questionnaire. Feedback from the pretest informed necessary revisions to enhance accuracy and ease of understanding for respondents.

To ensure high data quality throughout the study, supervisors carried out random checks of completed questionnaires to verify adherence to the study protocol and maintain consistency and accuracy in data collection.

### Data analysis and processing

All collected data were reviewed for completeness, accuracy, and consistency prior to analysis. Data cleaning was conducted to address any missing or inconsistent entries. The cleaned dataset was then analyzed using SPSS version 25. Descriptive statistics were used to summarize participants’ background characteristics. To assess associations between the dependent variable (neonatal near miss) and independent variables, Chi-square tests were employed. Variables showing a statistically significant association (*p* < 0.05) in the Chi-square test were included in a binary logistic regression model to identify independent predictors of neonatal near-miss events. The strength of associations was quantified using adjusted odds ratios (aORs) with 95% confidence intervals (CIs). A *p*-value of less than 0.05 was considered statistically significant in the final model.

### Ethical considerations

The study received ethical clearance from the Research Ethics Committee of SOS College of Health Science (Reference: SOSCHS/REC/2025/015), underscoring adherence to the highest standards of ethical conduct and research integrity. Informed consent was obtained from all participants before data collection, with strict measures in place to safeguard their privacy and confidentiality. Participation was fully voluntary, and individuals retained the right to withdraw from the study at any point without any form of penalty or disadvantage.

## Results

### Socio-demographic characteristics

The study included 973 participants, with 243 neonatal near miss (NNM) cases and 730 controls. Most participants (85%) lived in urban areas, though a higher proportion of cases (24.7%) were from rural areas compared to controls (11.8%). Maternal age was similar across groups, with over half of mothers under 25 years. The majority of mothers were married (96.3%), but unmarried mothers were slightly more common among cases (7%) than controls (2.6%).

A large portion of mothers lacked formal education (64.9%), with this proportion higher among cases (79.8%) than controls (59.9%). Similarly, more fathers of cases had no formal education (81.1%) compared to controls (50%). Maternal employment status and family size were comparable between groups, with about 82% of mothers working and roughly 42% of families having fewer than five members. Regarding income, nearly half of all families earned less than 100 USD monthly, with a notably higher percentage among cases (69.1%) versus controls (40.1%) (Table 1).

**Table 1:** Socio-demographic characteristics of respondents

Variables		Neonatal Near Miss		Total *N* (%)	*P*-value
		Case *n* (%)	Control *n* (%)		
***Residence***					***0.000***
Urban		183 (75.3)	644 (88.2)	827 (85.0)	
Rural		60 (24.7)	86 (11.8)	146 (15.0)	
***Maternal Age***					***0.421***
< 25 years		131 (53.9)	383 (52.5)	514 (52.8)	
26–35 years		97 (39.9)	315 (43.2)	412 (42.3)	
> 35 years		15 (6.2)	32 (4.4)	47 (4.9)	
***Maternal Marital Status***					***0.002***
Married		226 (93.0)	711 (97.4)	937 (96.3)	
Unmarried		17 (7.0)	19 (2.6)	36 (3.7)	
***Maternal education***					***0.000***
No formal education		194 (79.8)	437 (59.9)	631 (64.9)	
Formal education		49 (20.2)	293 (40.1)	342 (35.1)	
**Paternal education**					***0.000***
No formal education		197 (81.1)	365 (50.0)	562 (57.8)	
Formal education		46 (18.9)	365 (50.0)	411 (42.2)	
**Maternal working status**					***0.993***
Working		199 (81.9)	598 (81.9)	797 (81.9)	
Not working		44 (18.1)	132 (18.1)	176 (18.1)	
**Family size**					***0.893***
< 5 Individuals		101 (41.6)	307 (42.1)	408 (41.9)	
≥ 5 Individuals		142 (58.4)	423 (57.9)	565 (58.1)	
**Monthly family income**					***0.000***
< 100 USD		168 (69.1)	293 (40.1)	461 (47.4)	
≥ 100 USD		75 (30.9)	437 (59.9)	512 (52.6)	

### Maternal obstetric history and newborn characteristics

The study also assessed various obstetric and neonatal variables in relation to neonatal near miss (NNM) outcomes. Regarding parity, a larger proportion of NNM cases (78.6%) had more than three children, compared to 57.8% of controls, highlighting higher parity among cases. Short birth intervals were more common in NNM cases, with 47.7% having intervals shorter than 2 years, compared to 26.6% of controls. Similarly, antenatal care (ANC) attendance was significantly lower in NNM cases (21.4%) than in controls (63.6%), indicating a notable gap in maternal healthcare utilization.

In terms of history of stillbirth, 21.0% of NNM cases had a previous stillbirth, compared to only 4.5% of controls. Obstetric complications were more prevalent among women whose neonates experienced a near-miss (34.2%) compared to those whose neonates did not (10.4%), indicating that such complications are more common in pregnancies resulting in near-miss neonatal events. Premature rupture of membranes (PROM) did not show a significant difference between the groups, with 17.3% of cases and 17.8% of controls affected.

Regarding gestational age, a higher proportion of NNM cases (32.9%) were preterm (< 37 weeks), compared to 17.1% of controls. In contrast, term births (37–41 weeks) were more common in controls (77.8%) than in cases (65.0%). Prolonged labor was more frequent in NNM cases (22.2%) compared to controls (7.4%), suggesting a link between prolonged labor and NNM.

Concerning place of delivery, a higher proportion of NNM cases (78.6%) delivered at home, compared to 51.2% of controls. No significant difference was found in the mode of delivery, with 62.6% of NNM cases and 66.3% of controls delivering vaginally. Chronic medical conditions were more common among NNM cases (69.1%) compared to controls (40.1%), suggesting that underlying health issues may contribute to NNM. In terms of baby sex, 65.8% of NNM cases had male babies, compared to 49.0% of controls. Finally, birth weight was significantly associated with NNM, as 45.3% of NNM cases had low birth weight, compared to only 8.2% of controls, with most controls (91.8%) having normal birth weight (Table 2).

**Table 2:** Maternal obstetric history and newborn characteristics

Variables		Neonatal Near Miss		Total *N* (%)	*P*-value
		Case *n* (%)	Control *n* (%)		
**Parity**					***0.000***
≤ 3		52 (21.4)	422 (57.8)	474 (48.7)	
> 3		191 (78.6)	308 (42.2)	499 (51.3)	
**Short birth interval**					***0.000***
Yes		116 (47.7)	194 (26.6)	310 (31.9)	
No		127 (52.3)	536 (73.4)	663 (68.1)	
**ANC attendance**					***0.000***
Yes		52 (21.4)	464 (63.6)	516 (53.0)	
No		191 (78.6)	266 (36.4)	457 (47.0)	
**History of Stillbirth**					***0.000***
Yes		51 (21.0)	33 (4.5)	84 (8.6)	
No		192 (79.0)	697 (95.5)	889 (91.4)	
**Obstetric Complications**					***0.000***
Yes		83 (34.2)	76 (10.4)	159 (16.3)	
No		160 (65.8)	654 (89.6)	814 (83.7)	
**Premature Rupture of Membranes**					***0.853***
Yes		42 (17.3)	130 (17.8)	172 (17.7)	
No		201 (82.7)	600 (82.2)	801 (82.3)	
**Gestational Age at Birth**					***0.000***
Preterm (< 37 weeks)		80 (32.9)	125 (17.1)	205 (21.1)	
Post-term (≥ 42 weeks)		5 (2.1)	37 (5.1)	42 (4.3)	
Term (37–41 weeks)		158 (65.0)	568 (77.8)	726 (74.6)	
**Prolonged labour**					***0.000***
Yes		54 (22.2)	54 (7.4)	108 (11.1)	
No		189 (77.8)	676 (92.6)	865 (88.9)	
**Place of Delivery**					***0.000***
Health Facility		52 (21.4)	356 (48.8)	408 (41.9)	
Home		191 (78.6)	374 (51.2)	565 (58.1)	
**Mode of Delivery**					***0.287***
SVD		152 (62.6)	484 (66.3)	636 (65.4)	
Non SVD		91 (37.4)	246 (33.7)	337 (34.6)	
**Chronic Medical Conditions**					***0.000***
Yes		168 (69.1)	293 (40.1)	461 (47.4)	
No		75 (30.9)	437 (59.9)	512 (52.6)	
**Baby sex**					***0.000***
Male		160 (65.8)	372 (51.0)	532 (54.7)	
Female		83 (32.2)	358 (49.0)	441 (45.3)	
**Birth weight**					***0.000***
Low birth weight		110 (45.3)	60 (8.2)	170 (17.5)	
Normal birth weight		133 (54.7)	670 (91.8)	803 (82.5)	

### Determinants of neonatal near miss identified through multivariable analysis

Variables that showed statistical significance in the chi-square analysis were further examined using multivariable logistic regression. The multivariable analysis identified several factors significantly associated with neonatal near miss. These included maternal and paternal education, low monthly family income, high parity, short birth interval, lack of antenatal care (ANC) attendance, history of stillbirth, obstetric complications, non-term gestational age, prolonged labor, place of delivery, chronic medical conditions, male sex of the newborn, and low birth weight. In contrast, variables such as residence did not show a statistically significant association with neonatal near miss after adjusting for potential confounders.

Mothers without formal education had higher odds of neonatal near miss compared to those with formal education (AOR: 2.61; 95% CI: 2.004–2.412). Likewise, paternal lack of formal education was significantly associated with near miss events (aOR: 3.64; 95% CI: 2.448–5.419). Similarly, families with a monthly income of less than 100 USD were nearly three times more likely to experience a neonatal near miss (AOR: 2.82; 95% CI: 1.968–4.046). Having more than three children was associated with increased risk, while having three or fewer children was protective (aOR: 0.26; 95% CI: 0.179–0.392). A short birth interval of less than 24 months also significantly raised the odds (AOR: 1.97; 95% CI: 1.349–2.854).

Notably, mothers who attended antenatal care (ANC) during their last pregnancy were approximately 6 times less likely to experience a neonatal near miss compared to those who did not attend (aOR: 0.16; 95% CI: 0.100–0.240), highlighting ANC as a strong protective factor. In contrast, mothers with a previous history of stillbirth had more than 4 times higher odds of experiencing a neonatal near miss (aOR: 4.35; 95% CI: 2.870–6.606). Similarly, the presence of obstetric complications significantly increased the odds by over fourfold (AOR: 4.46; 95% CI: 3.127–6.373). Additionally, neonates born outside the term gestational window (preterm or post-term) were nearly 2 times more likely to experience a near miss compared to those born at term (AOR: 1.89; 95% CI: 1.375–2.588). On the other hand, prolonged labor was associated with a more than 3.5-fold increase in risk (aOR: 3.58; 95% CI: 2.373–5.391), underscoring the importance of timely obstetric care.

Delivering at a health facility significantly reduced the odds of experiencing a neonatal near miss by approximately five times (AOR: 0.21; 95% CI: 0.053–0.817), underscoring the protective role of institutional delivery. In contrast, mothers with chronic medical conditions had more than three times higher odds of experiencing a neonatal near miss compared to those without such conditions (aOR: 3.37; 95% CI: 4.484–12.120), indicating a strong association between maternal health status and neonatal outcomes. Moreover, male neonates were nearly twice as likely to be classified as near miss cases compared to females (aOR: 1.86; 95% CI: 1.371–2.510). Above all, low birth weight was the most significant predictor of neonatal near miss, with affected infants being over nine times more likely to experience life-threatening complications than those with normal birth weight (aOR: 9.34; 95% CI: 6.408–13.310) (Table 3).

**Table 3:** Factors associated with neonatal near miss among neonates

Characteristics	*N* (%)	Neonatal Near miss		AOR 95%(CI)
		Yes = 243	No = 730	
**Residence**				
Urban	827 (85.0)	183 (75.3)	644 (88.2)	1.24 (0.714–2.158)
Rural	146 (15.0)	60 (24.7)	86 (11.8)	1
**Marital status**				
Married	937 (96.3)	226 (93.0)	711 (97.4)	2.59 (0.978–6.863)
Unmarried	36 (3.7)	17 (7.0)	19 (2.60)	1
**Maternal education**				
No formal education	631 (64.9)	194 (79.8)	437 (59.9)	2.61 (2.004–2.412)*
Formal education	342 (35.1)	49 (20.2)	293 (40.1)	1
**Paternal education**				
No formal education	562 (57.8)	197 (81.1)	365 (50.0)	3.64 (2.448–5.419)*
Formal education	411 (42.2)	46 (18.9)	365 (50.0)	1
**Monthly family income**				
< 100 USD	461 (47.4)	168 (69.1)	293 (40.1)	2.82 (1.968–4.046)*
≥ 100 USD	512 (52.6)	75 (30.9)	437 (59.9)	1
**Parity**				
≤ 3	474 (48.7)	52 (21.4)	422 (57.8)	0.26 (0.179–0.392)*
> 3	499 (51.3)	191 (78.6)	308 (42.2)	1
**Short birth interval (< 24 Months)**				
Yes	310 (31.9)	116 (47.7)	194 (26.6)	1.97 (1.349–2.854)*
No	663 (68.1)	127 (52.3)	536 (73.4)	1
**ANC attendance for last pregnancy**				
Yes	516 (53.0)	52 (21.4)	464 (63.6)	0.16 (0.100–0.240)*
No	457 (47.0)	191 (78.6)	266 (36.4)	1
**History of Stillbirth**				
Yes	84 (8.6)	51 (21.0)	33 (4.5)	4.35 (2.870–6.606)*
No	889 (91.4)	192 (79.0)	697 (95.5)	1
**Obstetric Complications**				
Yes	159 (16.3)	83 (34.2)	76 (10.4)	4.46 (3.127–6.373)*
No	814 (83.7)	160 (65.8)	654 (89.6)	1
**Gestational Age at Birth**				
Non-term (pre & post)	247 (25.4)	85 (35.0)	162 (22.2)	1.89 (1.375–2.588)*
Term (37–41 weeks)	726 (74.6)	158 (65.0)	568 (77.8)	1
**Prolonged labour**				
Yes	108 (11.1)	54 (22.2)	54 (7.4)	3.58 (2.373–5.391)*
No	865 (88.9)	189 (77.8)	676 (92.6)	1
**Place of Delivery**				
Health Facility	52 (21.4)	356 (48.8)	408 (41.9)	0.21 (0.053–0.817)*
Home	191 (78.6)	374 (51.2)	565 (58.1)	1
**Chronic Medical Conditions**				
Yes	78 (8.0)	52 (21.4)	26 (3.60)	3.37 (4.484–12.120)*
No	895 (92.0)	191 (78.6)	704 (96.4)	1
**Baby sex**				
Male	532 (54.7)	372 (51.0)	532 (54.7)	1.86 (1.371–2.510)*
Female	441 (45.3)	358 (49.0)	441 (45.3)	1
**Birth weight**				
Low birth weight	170 (17.5)	110 (45.3)	60 (8.2)	9.34 (6.408–13.310)*
Normal birth weight	803 (82.5)	133 (54.7)	670 (91.8)	1

## Discussion

The study examined the determinants of neonatal near miss among neonates born or admitted to SOS Mother & Child Hospital in Mogadishu. The findings revealed that neonatal near miss remains a significant public health concern, influenced by a range of maternal, socioeconomic, obstetric, and neonatal factors. Key determinants identified in this study include lack of maternal and paternal formal education, low household income, high parity, short birth interval, lack of antenatal care (ANC) attendance, history of stillbirth, obstetric complications, non-term gestational age, prolonged labor, home delivery, chronic maternal medical conditions, male sex of the newborn, and low birth weight.

Mothers who attended ANC were approximately 6 times less likely to experience a neonatal near miss. A similar protective effect was reported in Ethiopia, where ANC attendance reduced the odds of neonatal near miss by 73%^23^. This could be explained by the fact that antenatal care provides an essential opportunity to identify and manage maternal and fetal complications early, promote birth preparedness, and ensure timely referral for high-risk pregnancies. The study also reported that low birth weight increased the odds of neonatal near miss more than 9-fold. This aligns with studies in India, where low birth weight was a key criterion for NNM [24]. This might be due to the fact that low birth weight neonates often suffer from immature organ development, reduced immunity, and poor thermoregulation, making them more susceptible to life-threatening conditions such as infections, respiratory distress, and feeding difficulties—especially in low-resource settings where access to neonatal intensive care may be limited [25].

Regarding the institutional delivery, the study found out that health facility delivery reduced the odds of neonatal near miss by about five times. This can be explained by the fact that institutional deliveries are attended by skilled health professionals who are equipped to manage labor complications, provide timely neonatal resuscitation, and ensure immediate postnatal care [26]. In line with this, the study found that women who experienced obstetric complications had a 4.5-fold increased risk of neonatal near miss compared to those without such complications [27]. More specifically, prolonged labor—one of the most common obstetric complications—was associated with over a 3.5-fold increase in the odds of neonatal near miss. Almost similar findings were reported in a study conducted in Ethiopia, where obstetric complications were significantly associated with increased odds of neonatal near miss [23].

With respect to gestational age, non-term neonates (preterm and post-term) had 1.9 times higher odds of neonatal near miss. In the light of the fact that preterm infants are more vulnerable to complications such as respiratory distress, hypothermia, and infections due to organ immaturity, while post-term births are often associated with increased risk of birth asphyxia, meconium aspiration, and placental insufficiency—all of which contribute to adverse neonatal outcomes. Studies in India and Ghana identified non-term gestational age as a critical criterion for neonatal near miss. These studies highlighted that both preterm and post-term births are associated with increased risks of severe neonatal complications [24, 28].

It was shown that chronic maternal conditions (e.g., anemia, hypertension) were linked to 3.4 times higher odds of neonatal near miss (NNM). A similar study conducted in India found that 74.5% of NNM cases involved maternal comorbidities [24]. This is attributable to chronic maternal health issues, such as hypertension, diabetes, and anemia, can significantly impair the physiological processes necessary for a healthy pregnancy.

Finally, regarding the sex of the newborn, the study revealed that male neonates had nearly double the odds of experiencing a neonatal near miss compared to females. This can be explained by the biological vulnerability of male infants, who are more prone to respiratory distress, infections, and slower lung maturation during the neonatal period. Although not conclusively evidenced by this study, existing literature suggests that male neonates generally have a higher risk of adverse outcomes due to these physiological factors [29, 30].

### Strengths and limitations

A key strength of this study is the use of standardized neonatal near miss criteria based on internationally recognized CLAP guidelines, which ensured consistency in case identification. The relatively large sample size and use of both structured interviews and medical record reviews enhanced the reliability of the findings. Additionally, the multivariable analysis allowed for adjustment of confounding variables, strengthening the validity of the associations identified.

Despite its valuable contributions, the study has certain limitations. Although it included a relatively large sample size, it was conducted in a single hospital, which may not capture the full variability of neonatal care practices. In addition, some variables were based on maternal recall, which may have introduced recall bias and affected the precision of the data collected.

### Conclusion & recommendation

The burden of neonatal near miss remains a critical public health concern in Somalia. This study identified several factors significantly associated with neonatal near miss among neonates in Mogadishu. These included lack of formal maternal and paternal education, low household income, high parity, short birth interval, absence of antenatal care (ANC), history of stillbirth, obstetric complications, non-term gestational age, prolonged labor, home delivery, maternal chronic illnesses, male sex of the newborn, and low birth weight.

Addressing these factors is essential to reducing neonatal morbidity and improving survival. Interventions that promote ANC attendance, safe delivery practices, maternal education, and early identification of high-risk pregnancies should be prioritized by both governmental and humanitarian actors. Strengthening facility-based care and improving access to skilled birth attendants can play a pivotal role in preventing life-threatening neonatal complications and achieving better outcomes for newborns in resource-limited settings.

## Data Availability

No datasets were generated or analysed during the current study.
